# Factors that Influence 2-Year Progression-Free Survival Among Head and Neck Cancer Patients

**DOI:** 10.1007/s44197-021-00016-2

**Published:** 2021-11-30

**Authors:** Cosphiadi Irawan, Larangga Gempa Benbella, Andhika Rachman, Arif Mansjoer

**Affiliations:** 1 Hematology and Medical Oncology Division, Department of Internal Medicine, Faculty of Medicine, Universitas Indonesia-Cipto Mangunkusumo National Central General Hospital (RSCM), Jl. Diponegoro no. 71, Jakarta, 10430 Indonesia; 2 Department of Internal Medicine, Faculty of Medicine, Universitas Indonesia-Cipto Mangunkusumo National Central General Hospital (RSCM), Jakarta, Indonesia; 3 Cardiology Division, Department of Internal Medicine, Faculty of Medicine, Universitas Indonesia-Cipto Mangunkusumo National Central General Hospital (RSCM), Jakarta, Indonesia

**Keywords:** Head and neck cancer, Progression-free survival, Factor, Mortality

## Abstract

**Objectives:**

The majority of patients with head and neck cancer (HNC) come to the hospital at advanced stages. This research was conducted to determine the mortality, 2-year progression-free survival (PFS) and factors that influenced PFS of HNC patients.

**Methods:**

A retrospective cohort study was conducted among locally advanced HNC patients who underwent chemoradiation for the first time at RSCM from January 2015 to December 2017. Data were retrieved through medical records. Laboratory data were taken 2–4 weeks prior and 2–4 weeks after chemoradiation. PFS observation started from the first day of chemoradiation until disease progression or death. PFS data were recorded in two groups: ≤ 2 years and > 2 years. The Chi-square test was used for bivariate analysis with the Fischer-exact test as an alternative. Variables will be further tested using multivariate logistic regression tests.

**Results:**

Among 216 subjects, there were 103 (47.69%) patients who did not reach overall survival (OS) > 2 years. There were 108 (50%) patients who had PFS > 2 years. Based on the results of multivariate analysis, it was found that smoking, hemoglobin level ≤ 12 g/dl, ECOG (Eastern Cooperative Oncology Group) 1–2, and negative therapeutic response were associated with poor PFS. Hazard ratio (HR) for 2-year PFS for Brinkman index > 250 was 1.36 (95% CI 0.93–2.00; *p* = 0.02); HR for Hb ≤ 12 g/dl was 1.65 (95% CI 1.13–2.42; *p* = 0.01); HR for ECOG 1–2 was 4.05 (95% CI 1.49–11.00; *p* < 0.01); and HR for negative therapeutic response was 2.37 (95% CI 1.43–3.94; *p* < 0.01).

**Conclusion:**

Mortality of HNC patients within 2 years is 47.69%, with a 2-year PFS reaching 50%. Cigarette smoking, low hemoglobin levels, poor performance status, and negative therapeutic response (non-responders) negatively affect the 2-year PFS.

## Introduction

Head and neck cancer (HNC) is the sixth most common cancer in the world [1]. The incidence of HNCs is increasing every 5 years [2]. There are about seven hundred thousand new patients in which four hundred thousand people died every year. The majority of HNC cancer patients who come to the hospital are at locally advanced stage. The proportion of those with stage III reaches 24–25% and stage IV 44–54% [3, 4].

Progression-free survival (PFS) and overall survival (OS) are useful outcome measures in evaluating HNC patients. The advantage of PFS over OS is that it can evaluate survival and treatment [5, 6]. PFS is defined as the time from the treatment starts until the patient experiences disease progression or death from any cause. Several studies have shown that various factors can affect 2-year PFS.

Espeli, et al. [7] found that smoking is the significant and independent factor for PFS in locally advanced HNC. Smoking has been known to exert negative impact on the carcinogenesis and cancer outcome. However, regional variations in tobacco products and smoking practice might contribute to different risk profiles in HNC patients. Hemoglobin level also influences the progressivity and survival of cancer. Hypoxia that occurs within tumor microenvironment induces sustained proliferation [8].

Szturz et al. [9] reported that patient with age > 75 years has worse survival compared to age<75 years. Moreover, performance status is a crucial aspect that has to be considered in a cancer patient [10]. Finally, the presence of comorbidities influences the survival of cancer [11]. Current findings regarding the role of comorbidities and PFS in HNC patients showed conflicting results [12, 13]. Other factors that were considered to influence 2-year PFS in HNC patients include neutrophil-to-lymphocyte ratio and platelet-to-lymphocyte ratio [6], renal function [14, 15], and therapeutic response [16, 17].

Since the aforementioned factors have not been extensively studied in relation to 2-year PFS, the authors want to identify which factors significantly affect 2-year PFS. Moreover, research related to HNC in Indonesia is relatively few. For this reason, this study aims to determine the 2-year mortality of HNC patients, determine the proportion of 2-year PFS, and assess the factors that affect 2-year PFS in HNC patients.

## Methods

A retrospective cohort study was conducted to examine the factors that affect 2-year PFS. Sample size calculation was done using the hypothesis test formula for 2 independent proportions with a minimum sample size of 188. Sampling was done consecutively with inclusion criteria that were patients with locally advanced head and neck cancer undergoing chemoradiation at Cipto Mangunkusumo National Central General Hospital from January 2015 to December 2017 and aged ≥ 18 years. Data were obtained from medical records. Locally advanced HNC includes stages III, IVa, and IVb of HNC. This staging is based on TNM Staging of Head and Neck Cancer and Neck Dissection Classification from American Joint Commission on Cancer (AJCC) Cancer Staging Manual, which uses histopathology and imaging examination. Criteria for exclusion were patients who had chemotherapy or chemoradiation in hospitals other than Cipto Mangunkusumo National Central General Hospital, undergoing full-dose chemotherapy or Neo-Adjuvant Chemotherapy (NAC), the medical record was not found/burned/ retained, and the patient could not be contacted. Locally advanced HNC patients who did not complete chemoradiation therapy are categorized as dropouts.

Variables collected were included consumption of salted fish, age, performance status, amount of cigarette smoking by Brinkman index which is defined as number of daily number of cigarettes times years of smoking duration, head and neck Charlson comorbidity index (HN-CCI), estimated glomerular filtration rate (eGFR), hemoglobin, body mass index (BMI), albumin, neutrophil-to-lymphocyte ratio (NLR), platelet-to-lymphocyte ratio (PLR), HNC histology, and therapeutic response using RECIST criteria. Therapeutic responses were defined by RECIST criteria and were categorized into responders (Complete Response and Partial Response) and non-responders (Stable Disease and Progressive Disease).

Bivariate analysis was done by the Chi-square test or Fisher’s exact test if the Chi-square test requirements are not met, and the multivariate analysis uses logistic regression. Kaplan–Meier method was used in the survival data analysis. Those analysis were performed using Stata^®^ Program.

## Results

**Fig. 1:**
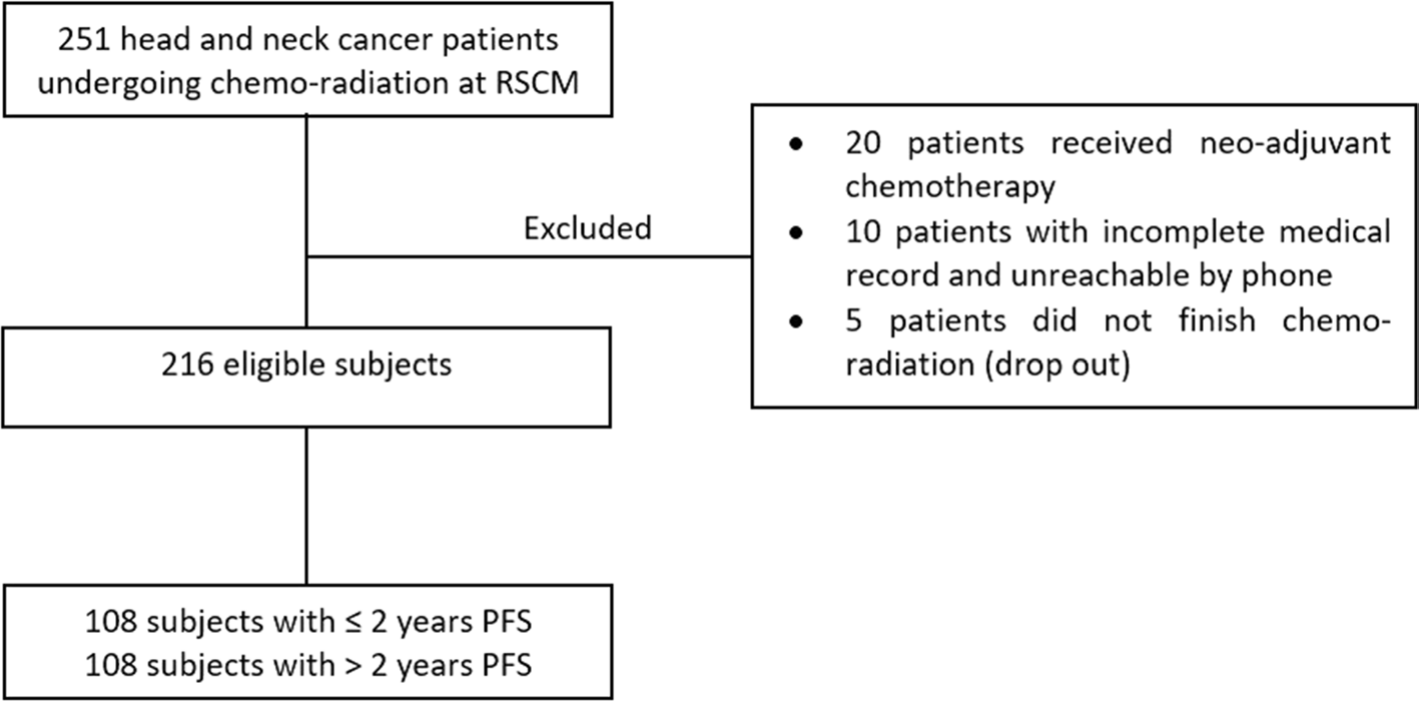
Study enrollment flow diagram. PFS: progression-free survival

There were 251 HNC patients who underwent chemoradiation at the Cipto Mangunkusumo National Central General Hospital and fulfilled the inclusion criteria. However, 45 patients met dropped out due to incomplete radiation schedules. This study included 216 eligible subjects (Fig. 1).

The majority of HNC patients are 56–65 years old (27.31%) with a mean age of 50 years and a standard deviation of 13.06. The proportion of male to female subjects was 3:1. Most of the HNC patients come from the Javanese, Sundanese, Batak and Betawi ethnicity. The characteristics of HNC patients can be seen in Table 1.

**Table 1:** Characteristics of HNC patients in Cipto Mangunkusumo National Central General Hospital from January 2015 to December 2017

Characteristic	*n* = 216
Age in years, *n* (%)	
18–25	14 (6.48)
26–35	13 (6.02)
36–45	51 (23.61)
46–55	57 (26.39)
56–65	59 (27.31)
> 65	22 (10.19)
Mean age ± SD	50 ± 13.06
Gender, *n* (%)	
Male	165 (76.39)
Female	51 (23.61)
Cancer location, *n* (%)	
Larynx	38 (17.59)
Oropharynx	12 (5.56)
Nasopharynx	155 (71.76)
Sino nasal	12 (5.56)
PFS, *n* (%)	
> 2 years	108 (50)
≤ 2 years	108 (50)
2-year mortality, *n* (%)	
No (survived)	113 (52.31)
Yes (died)	103 (47.69)
HN-CCI (head and neck Charlson comorbidity index)	
0	156 (72.2)
1	39 (18.06)
2	16 (7.41)
3	4 (1.85)
4	1 (0.46)
Hemoglobin, *n* (%)	
> 12 g/dl	139 (64.35)
≤ 12 g dl	77 (35.65)
Albumin, *n* (%)	
> 3.5 g/dl	92 (81.42)
≤ 3.5 g/dl	21 (18.58)
Albumin (g/dL), mean SD	4,01 0,51
BMI, *n* (%)	
> 18.5 kg/m2	176 (81.48)
≤ 18.5 kg/m2	40 (18.52)
ECOG	
0	24 (11.11)
1	156 (72.22)
2	36 (16.67)
NLR, *n* (%)	
≤ 2	43 (19.91)
> 2	173 (80.09)
PLR, *n* (%)	
≤ 100	21 (9.72)
> 100	195 (90.28)
eGFR, *n* (%)	
> 60 ml/min/1.73m^2^	205 (94.91)
≤ 60 ml/min/1.73m^2^	11 (5.09)
Smoking, *n* (%)	
Brinkman index ≤ 250	100 (46.30)
Brinkman index > 250	116 (53.70)
Consumption of salted fish, *n* (%)	
≤ 5 times during lifetime	62 (28.70)
> 5 times during lifetime	154 (71.30)
Duration of salted fish consumption, *n* (%)	
≤ 10 years	72 (33.33)
> 10 years	144 (66.67)
Stage	
III	47 (21.76)
IVA	142 (65.74)
IVB	27 (12.50)
Keratinized squamous cell	
No	176 (81.48)
Yes	40 (18.52)
Therapeutics response	
Responsive	195 (90.28)
Unresponsive	21 (9.72)
Radiotherapy plane	
IMRT	115 (53.24)
2D/3D	101 (46.76)
Evaluation accuracy, *n* (%)	
Yes	6 (2.78)
No	210 (97.22)

*PFS* Progression-free survival, *SD* standard deviation, *BMI* body mass index, *ECOG* Eastern Cooperative Oncology Group, *NLR* neutrophil-to-lymphocyte ratio, *PLR* platelet-to-lymphocyte ratio, *eGFR* estimated glomerular filtration rate, *IMRT* intensity modulated radiation therapy

**Fig. 2:**
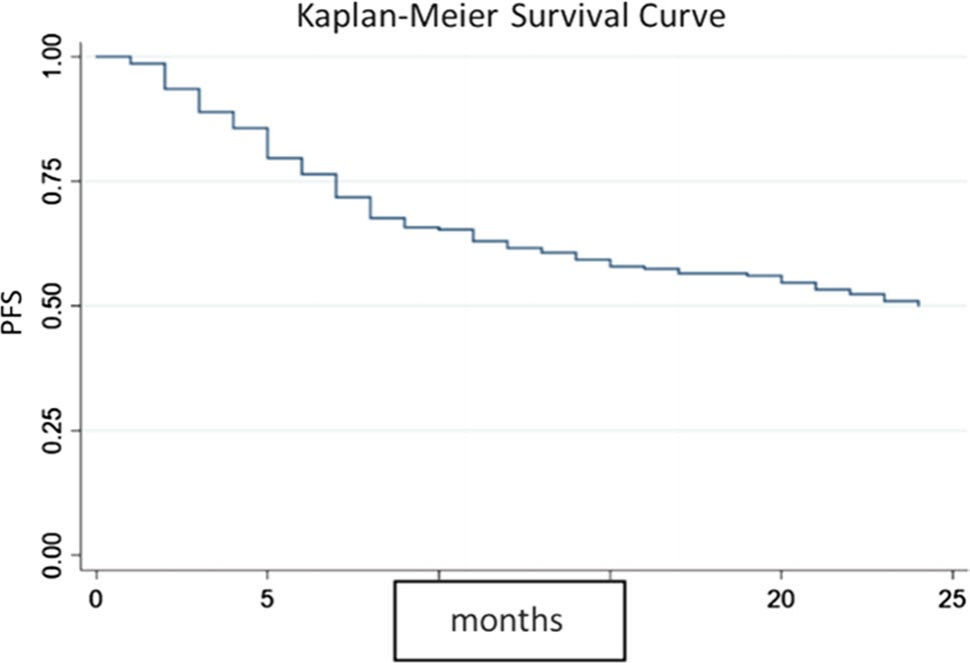
Kaplan–Meier curves for 2-year PFS of HNC patients. *PFS* progression-free survival, *HNC* head and neck cancer

As many as 47.69% of local HNC patients died within 2 years, while there were around 50% who reached 2-year PFS. Figure 2 shows Kaplan–Meier survival curve of 2-year PFS among the study subjects. In bivariate analysis, factors that significantly influence PFS were hemoglobin level ≤ 12 g/dl (*p* = 0.02), ECOG 1–2 (*p* <0.01), albumin level ≤ 3.5 g/dl (*p* <0.01), Brinkman index> 250 (*p* = 0.05), and the therapeutic response (*p* < 0.01). Some other variables that are eligible for multivariate analysis (*p* < 0.25) were NLR> 2 (*p* = 0.23), eGFR≤ 60 ml/min/1.73 m^2^ (*p* = 0.11), and the stage of cancer (*p* = 0.10). The complete bivariate analysis results can be seen in Table 2.

**Table 2:** Bivariate analysis of factors associated with 2-year PFS in HNC patients

	PFS		*p*
	≤ 2 years (*n* = 108)	> 2 years (*n* = 108)	
Age, year *n* (%)			
≤ 60	85 (78.70%)	81 (75%)	0.519
> 60	23 (21.30%)	27 (25%)	
Comorbidity			
No comorbidities	79 (73.51%)	77 (71.30%)	0.761
With comorbidities	29 (26.80%)	79 (28.70%)	
Hemoglobin			
> 12 g/dl	61 (56.48%)	78 (72.22%)	0.0157
≤ 12 g/dl	47 (43.53%)	30 (27.78%)	
Albumin			
> 3.5 g/dl	41 (70.69%)	51 (92.73%)	0.0023
≤ 3.5 g/dl	17 (29.31%)	4 (7.27%)	
BMI			
> 18.5 kg/m^2^	85 (78.70%)	91 (84.26%)	0.29
≤ 18.5 kg/m^2^	23 (21.30%)	17 (15.74%)	
ECOG			
0	4 (3.70%)	20 (18.52%)	0.0004
1–2	104 (96.30%)	88 (81.48%)	
NLR			
≤ 2	18 (16.67%)	25 (23.15%)	0.233
> 2	90 (83.33%)	83 (76.85%)	
PLR			
≤ 100	9 (8.33%)	13 (12.04%)	0.368
> 100	99 (91.67%)	95 (87.96%)	
eGFR			
> 60 ml/min/1.73m2	105 (97.23%)	100 (92.60%)	0.121
≤ 60 ml/min/1.73m2	3 (2.77%)	8 (7.40%)	
Brinkman index			
≤ 250	43 (39.81%)	57 (52.78%)	0.05
> 250	65 (60.19%)	51 (47.22%)	
Consumption of salted fish, *n* (%)			
≤ 5 times during lifetime	31 (28.70%)	31 (28.70%)	1
> 5 times during lifetime	77 (71.30%)	77 (71.30%)	
Duration of salted fish consumption, *n* (%)			
≤ 10 years	35 (32.41%)	37 (34.26%)	0.77
> 10 years	73 (67.59%)	71 (65.74%)	
Stage			
III	18 (16.67%)	29 (26.85%)	0.0697
IVA–IVB	90 (83.33%)	79 (73.15%)	
Keratinized squamous cell			
No	88 (81.48%)	88 (81.48%)	1
Yes	20 (18.52%)	20 (18.52%)	
Therapeutics response			
Responsive	90 (83.33%)	105 (97.22%)	0.0006
Unresponsive	18 (16.67%)	3 (2.78%)	
Still smoking			
No	61 (56.48%)	81 (75%)	0.0041
Yes	47 (43.52%)	27 (25%)	

*PFS* Progression-free survival, *SD* standard deviation, *BMI* body mass index, *ECOG*: Eastern Cooperative Oncology Group, *NLR* neutrophil-to-lymphocyte ratio, *PLR* platelet-to-lymphocyte ratio, *eGFR* estimated glomerular filtration rate, *IMRT* intensity modulated radiation therapy

**Fig. 3:**
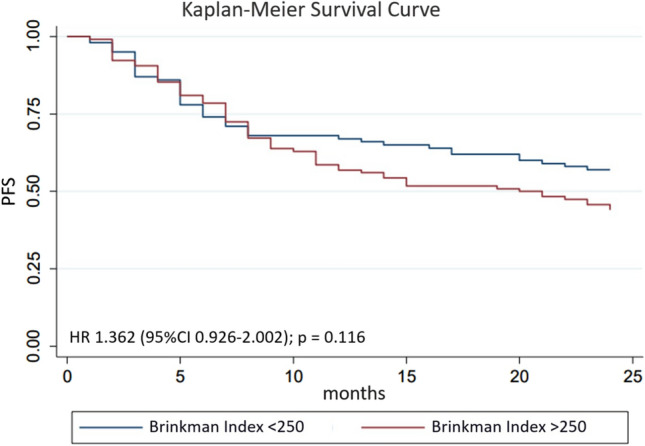
Kaplan–Meier curves for 2-year PFS of HNC patients with Brinkman index ≤ 250 and Brinkman index > 250. *PFS* progression-free survival, *HNC* head and neck cancer

**Fig. 4:**
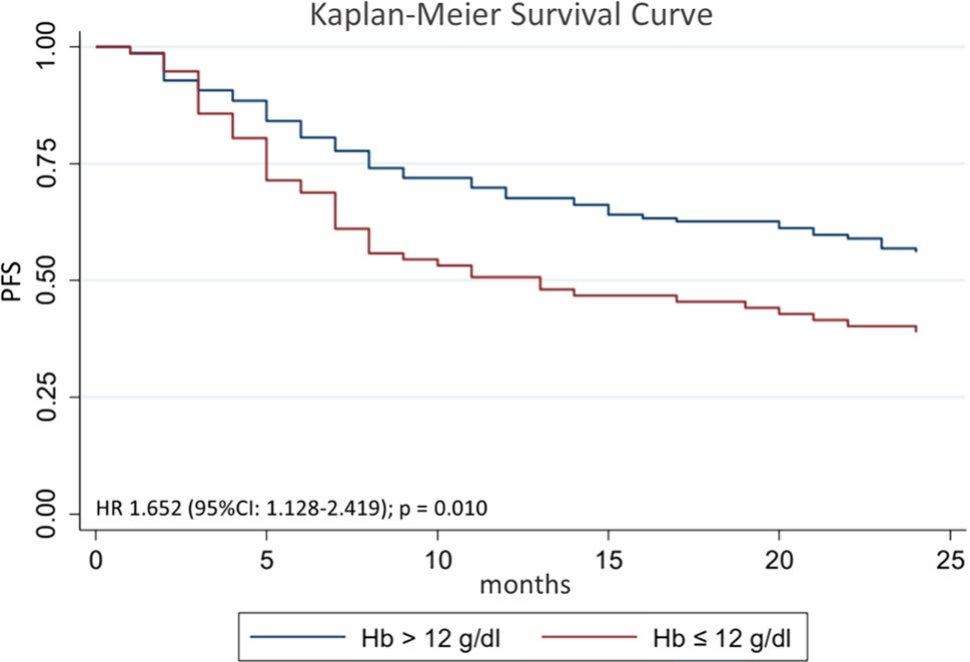
Kaplan–Meier curves for 2-year PFS of HNC patients with Hb > 12 g/dl and Hb ≤ 12 g/dl. *PFS* progression-free survival, *HNC* head and neck cancer; Hb: hemoglobin

**Fig. 5:**
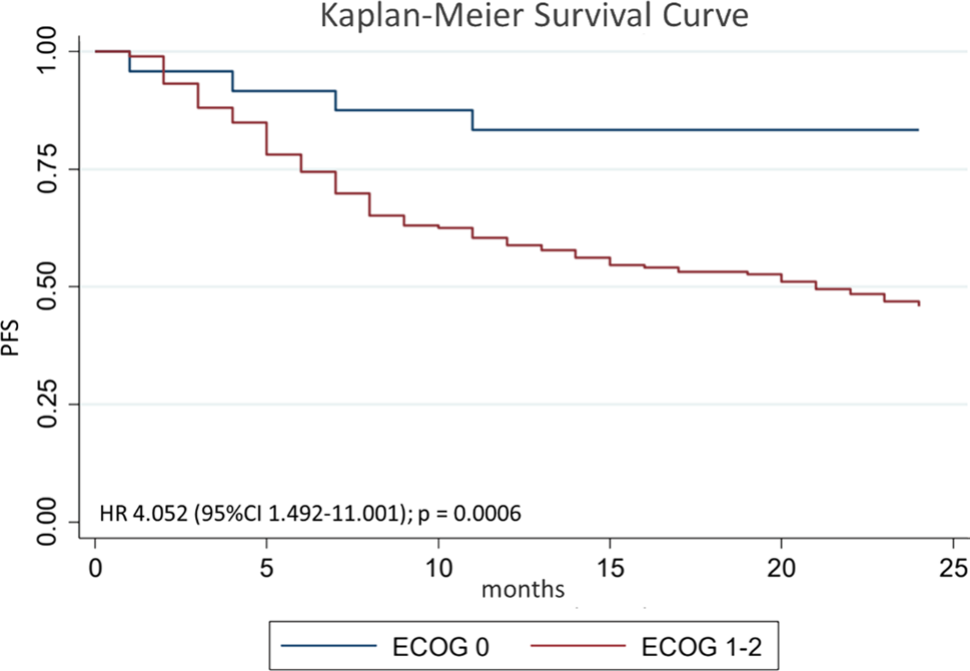
Kaplan–Meier curves for 2-year PFS of HNC patients with ECOG 0 and ECOG 1–2. *PFS* progression-free survival, *HNC* head and neck cancer, *ECOG* Eastern Cooperative Oncology Group

**Fig. 6:**
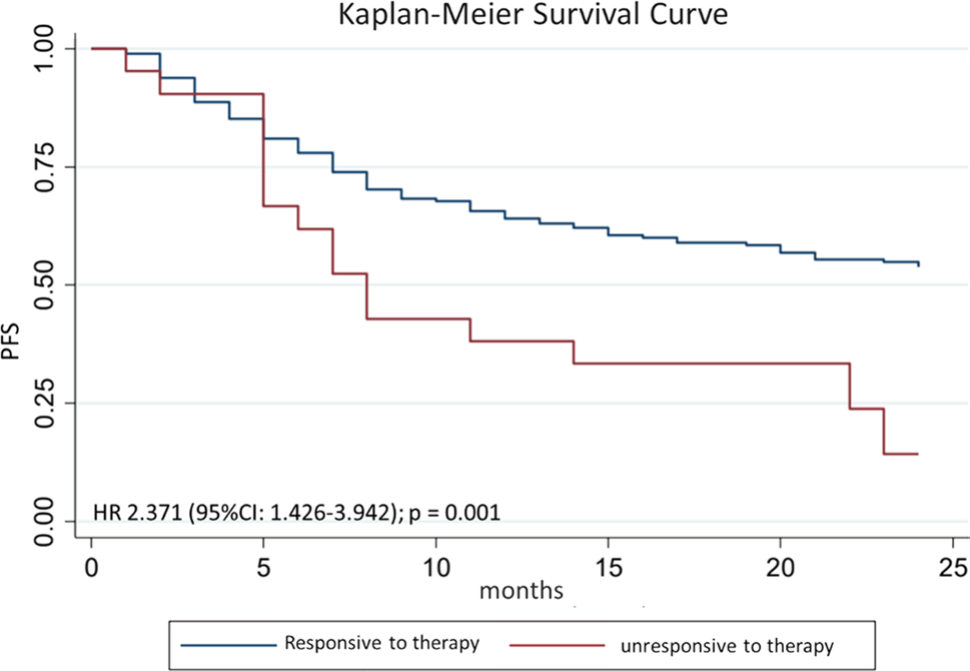
Kaplan–Meier curves for 2-year PFS of HNC patients with responsive and unresponsive to therapy. PFS: progression-free survival; *HNC* head and neck cancer

After multivariate analysis, the factors that influence 2-year PFS were smoking with Brinkman index> 250 (*p* = 0.02), hemoglobin level ≤ 12 g/dl (*p* < 0.01), ECOG 1–2 (*p* = 0.02), and therapeutic response (*p* < 0.01). Complete multivariate analysis can be seen in Table 3. Hazard ratio (HR) for 2-year PFS for Brinkman index>250 was 1.36 (95% CI 0.93–2.00; *p* = 0.02; Figure 3); HR for Hb ≤ 12 g/dl was 1.65 (95% CI 1.13–2.42; *p* = 0.01; Figure 4); HR for ECOG 1–2 was 4.05 (95% CI 1.49–11.00; *p* < 0.01; Figure 5); and HR for negative therapeutic response was 2.37 (95% CI 1.43–3.94; *p* < 0.01; Figure 6).

**Table 3:** Multivariate analysis of factors associated with 2-year PFS in HNC patients

Factors	*p*
Brinkman index > 250	0.028
Hemoglobin level below 12 g/dl	0.005
ECOG 1–2	0.003
Negative therapeutic response	0.002

*PFS* Progression-free survival, *HNC* head and neck cancer, *ECOG* Eastern Cooperative Oncology Group

## Discussion

This study found that 50% of patients reached 2-year PFS, with 47.69% died within 2 years. Factors that significantly affect 2-year PFS include cigarette smoking (Brinkman index > 250), hemoglobin level < 12 g/dl, ECOG 1–2, and absence of therapeutic response. Individuals who smoke are generally more susceptible to cancer. In this study, it was evident that smoking affected cancer progression (HR 1.36; 95% CI 0.93–2.00; *p* = 0.02). This finding is similar to Espelli et al. who found that cigarette smoking negatively affects 2-year PFS [7]. The mechanism of cigarettes smoking in tumor progression is known to be related to hypoxic mechanisms at the tissue level. Hypoxia triggers the formation of hypoxia inducible factor 1 (HIF-1). Overexpression of HIF-1 can stimulate vascular endothelial growth factor (VEGF) which promotes angiogenesis as well as the aggressiveness of the tumor [8].

Hemoglobin level ≤ 12 g/dl also negatively affects 2-year PFS progression (HR 1.65; 95% CI 1.13–2.42; *p* = 0.01). Our findings are similar to other studies conducted by Moon et al. [6] and Gorphe et al. [18]. Anemia plays an important role in decreasing intra-tissue oxygenation, reducing levels of free radicals, and reducing sensitivity to radiation therapy. Tissue hypoxia also causes mutations in p53 tumor suppressor genes which in turns trigger cancer progression and metastasis.

Performance status represented by ECOG in this study showed a significant effect on 2-year PFS, with hazard ratio for ECOG 1-2 4.05 (95% CI 1.49–11.00; *p* < 0.01). This is in line with the results obtained by Von de Grun et al. [18]. ECOG is a general picture of the patient’s overall performance status. Poor ECOG can affect the progression of cancer and eligibility to therapy. Cancer progression occurs more rapidly in patients with poor ECOG. In addition, ECOG can also describe the readiness of the patient’s physical status in receiving chemoradiation. In our study, there were no participants with ECOG > 2 who underwent chemoradiation in RSCM. In addition, hospital policy stated that patients with ECOG > 2 could not undergo chemotherapy and chemoradiation.

Therapeutic response also affects 2-year PFS in locally advanced HNC patients undergoing chemoradiation. Hazard ratio for negative therapeutic response was 2.37 (95% CI 1.43–3.94; *p* < 0.01). Research on the effect of therapeutic response to 2-year PFS in HNC patients is rarely conducted, but He Lijie et al. [17] found that the therapeutic response affected 5-year PFS in non-small cell lung cancer. This paper used response evaluation criteria in solid tumors (RECIST) criteria to evaluate therapeutic responses by dividing therapy response categories into responders (Complete Response and Partial Response) and non-responders (Stable Disease and Progressive Disease).

In this study, other factors that might contribute to 2-year PFS in HNC were also studied. Low albumin levels can be used as a predictor for the survival of cancer patients who underwent chemotherapy and radiation [19]. Our findings showed that it did not affect 2-year PFS. Moreover, 60% of the participants were>60 years old. Szturz et al. have reported that old age affects overall survival [9]. We did not found that age affects PFS (RR 0.90; 95% CI 0.64–1.25; *p* = 0.52). This result was consistent with previous studies from Pytynia et al. [20] and Fisher et al. [21]. A reasonable explanation for this was because most patients had no comorbidities and good performance status regardless of their age.

Comorbidities are believed to influence the survival of HNC patients [11, 12, 22, 23], but we did not find the difference between the presence of comorbidities using HN-CCI with PFS (RR 0.96; 95% CI 0.70–1.29; *p* = 0.76). Our results were consistent the results from study by with Peddi et al. [13] who reported that comorbidities did not influence PFS.

This study also found that BMI did not affect PFS (RR 1.2; 95% CI 0.86–1.62; *p* = 0.29). Moreover, NLR and PLR was not associated with PFS (RR 1.24; 95% CI 0.85–1.82; *p* = 0.23 for NLR and RR 0.53; 95% CI 0.2–1.41; *p* = 0.11 for PLR). We found that 83% of patients with NLR > 2 underwent 2-year progression and 76.85% of patients with NLR ≤ 2 underwent progression. The apparent lack of association between NLR and PLR with PFS could be explained by the normal range of neutrophil, lymphocyte, and thrombocyte in most of our study participants.

Our result showed that no correlation between renal function and 2-year PFS (RR 0.53; 95% CI 0.2–1.41; *p* = 0.11). These findings were in contrast with previous research reported in the literature [24–27]. We hypothesized that the lack of correlation is due to the majority (94%) of the participants had normal renal function with eGFR > 60 ml/min/1.73 m^2^. Therefore, it was difficult to detect any differences since the proportion of participants with poor renal function was less than 10%.

Salted fish consumption did not affect the PFS of HNC. Neither the amount nor duration of salted fish consumption was associated with 2-year PFS (RR 1.04; 95% CI 0.783–1.389; *p* = 0.77). These findings were in contradiction with previous results reported in the literature [28, 29]. Salted fish is one of the most common dishes commonly eaten by lower economic class society in Indonesia. Salted fish contains a carcinogenic substance such as NDMA (*N*-dimethyl-nitrosamine) [30]. Exposure to salted fish is associated with an increased risk of nasopharyngeal cancer up to 2.2 times compared to those not exposed to salted fish. Salted fish consumption is also known to affect the progression of nasopharyngeal cancer [31]. This apparent lack of correlation in our study can be attributed to the data collection method, which was food-recall. Moreover, information on the history of consumption of salted fish from HNC patients is sometimes obtained from the patient’s family, especially patients who have died. In addition, this study did not look at the process of cooking salted fish and measure the level of salt given during fish processing. Therefore, bias might occur and further study regarding this issue should address the quantity and quality of salted fish consumed more accurately.

This study also showed that staging of HNC had no significant effect on 2-year PFS (RR 1.39; 95% CI 0.94–2.05; *p* = 0.07). This is different from the results obtained by Ricketts et al. (32) who reported that cancer stage affected 2-year PFS. Furthermore, Rusthoven et al. [33] and Cadoni et al. [34] showed that cancer stage affected overall 3-year (*p* < 0.001) and 5-year [*p* = 0.008] survival. The cause of this discrepancy was due to the influence of other variables. Our data showed clinically significant difference in survival, although it was not statistically significant. Small sample size might contribute to this result. Moreover, longer follow-up might be needed to determine if cancer staging plays a role in survival. In this study, histology differentiation of keratinized and non-keratinized squamous cell cancer did not affect 2-year PFS. Although histologic subtypes were known to influence tumor biology, our study showed that in HNC patients, this classification does not affect 2-year PFS.

### Strengths and Limitations of Research

This study is the first study to look at 2-year PFS in HNC patients and influencing factors in Indonesia. This research is expected to be the beginning of the subsequent research related to HNCs. This study uses a retrospective cohort design. The limitations of this study are related to the limitations of data that can be taken as the shortcoming of retrospective cohorts in general, but we tried to fulfill these data by making telephone contact with patients and their families.

This study could benefit from the addition of the data regarding human papilloma virus (HPV), epidermal growth factor receptor (EGFR), lymphocytes percentage in tumoral tissue or hematologic factors such as lymphocytes, CD4, and CD8. Adelstein et al. reported that HPV, EBV, and staging were essential components to evaluate HNC patients’ progressivity and mortality. However, biomarker investigation such as EBV and HPV is not yet fully available in all regions in Indonesia and is still relatively expensive [35]. These markers should be considered in further research

## Conclusion

Mortality of HNC patients within 2 years is 47.69%, with a 2-year PFS reaching 50%. Cigarette smoking, low hemoglobin levels, poor performance status, and negative therapeutic response negatively affect the 2-year PFS.

## Data Availability

The data that support the findings of this study are available on request from the corresponding author.

## Abbreviations

BMI: Body mass index
ECOG: Eastern Cooperative Oncology Group
eGFR: Estimated glomerular filtration rate
HIF-1: Hypoxia inducible factor 1
HNC: Head and neck cancer
HN-CCI: Head and neck Charlson comorbidity index
NAC: Neo-adjuvant chemotherapy
NLR: Neutrophil-to-lymphocyte ratio
OS: Overall survival
PFS: Progression-free survival
PLR: Platelet-to-lymphocyte ratio
RECIST: Response evaluation criteria in solid tumors
RSCM: *Rumah* *Sakit* *Umum* *Pusat* *Nasional* *Cipto* *Mangunkusumo* (Cipto Mangunkusumo National Central General Hospital)
VEGF: Vascular endothelial growth factor
